# Experience and analysis of Delphian lymph node metastasis in patients with papillary thyroid carcinoma

**DOI:** 10.1186/1477-7819-10-226

**Published:** 2012-10-30

**Authors:** Won Woong Kim, Song I Yang, Jeong Hoon Kim, Young Sik Choi, Yo-Han Park, Su Kyoung Kwon

**Affiliations:** 1 Department of Surgery, Kosin University College of Medicine, 34 Amnam-dong, Seo-gu, Busan, Korea; 2 Department of Internal Medicine, Kosin University College of Medicine, 34 Amnam-dong, Seo-gu, Busan, Korea

**Keywords:** Delphian lymph node, Papillary thyroid cancer, Central neck lymph node

## Abstract

**Background:**

Recently, lymph node metastasis (LNM) has been regarded as an important factor influencing loco-regional recurrence and survival rate in papillary thyroid cancer (PTC) patients. The aims of this study were to investigate the detection rate and metastasis rate of the Delphian lymph node (DLN) and clinical patterns related to regional LNM, and to examine how DLN metastasis affects PTC treatment.

**Methods:**

We reviewed the medical records of 413 patients with pathologically confirmed PTC from among 452 patients who underwent thyroid surgery between January 2010 and October 2010 in the Department of Endocrine Surgery at Kosin University Gospel Hospital in Busan, South Korea.

**Results:**

Multivariate analyses revealed a significantly higher proportion of cases with lymphovascular invasion (56.6% vs. 12.5%, *P* <0.001), central neck node metastasis (88.6% vs. 34.5%, *P* <0.001) and lateral neck node metastasis (47.2% vs. 10.2%, *P* <0.005) among cases with DLN metastasis compared to those without. The negative predictive value (NPV) of DLN metastasis with regard to the presence of contralateral central LNM for cases with a tumor size 1 cm or smaller than 1 cm was found to be 93.3% (127/136).

**Conclusion:**

When DLN metastasis is not detected in papillary thyroid microcarcinomas (PTMC), thyroid lobectomy on the affected side and ipsilateral central neck lymph node dissection should be sufficient. In addition, even in cases where lateral neck LNM is not detected on preoperative examination, if DLN metastasis is detected postoperatively, more careful attention should be paid to the lateral neck nodes during follow-up.

## Background

Now the most commonly observed endocrine organ cancer is thyroid cancer [1,2]. Therefore, many studies have been performed on the surgical scope of thyroid cancer and on the treatment prognosis [1-4].

With papillary thyroid cancer (PTC), the effect of cervical lymph node metastasis (LNM) has been a less significant factor influencing the survival rate [5], but several recent studies reported that LNM has an effect on loco-regional recurrence and survival rate [1,3,6-11]. The development of ultrasonography that could be used before surgery made the evaluation of disease progression less complicated, and the use of frozen section biopsy in determining the scope of surgery intraoperatively, along with careful lymph node dissection, may lower the lymph node recurrence rate to improve the disease-free survival [12-14].

Level VI nodes are the most common nodal regions found to harbor metastasis and are made up of the Delphian (prelaryngeal), paratracheal and pretracheal nodes. Among Level VI nodes, the Delphian lymph node (DLN) sits directly anterior to the cricothyroid membrane between the cricothyroid muscles and is perhaps the most important of the level VI nodes surgically because it is the first lymph node encountered during total thyroidectomy [15,16]. Although debate remains on how lymph node metastasis progresses, it is common knowledge that the pretracheal lymph nodes are located in front of the isthmus to be drained out toward the mediastinum, and the DLNs are drained out toward the lateral neck following the superior thyroid artery [15-17]. Especially in laryngeal cancer, DLN metastasis is known as a predictor of extensive LNM, high recurrence rate and increased mortality rate. Therefore, it could serve as a useful parameter for determining the appropriate surgical treatment method, the need for additional treatment and prognosis. In thyroid cancer, however, due to the limited data on DLN metastasis, its clinical significance is still debated [15,16,18-21].

The aim of the present study was to determine whether the presence of DLN metastasis could be utilized in planning the surgical scope for thyroid cancer and selecting treatment options. We investigated the detection and metastasis rates and the associated clinical patterns of the Delphian lymph node in thyroid cancer, including the relationship with further metastasis in the central compartment.

### Patients and methods

We reviewed the medical records of 413 patients with pathologically confirmed PTC from among a total of 452 patients who underwent thyroid surgery between January and October 2010 at the Department of Endocrine Surgery of Kosin University Gospel Hospital in Busan, South Korea. We also gathered data on cervical LNM by the presence of DLN metastases.

After removing all tissues superior to thyroid isthmus and anterior to the cricothyroid membrane where the DLN are located, the presence of DLN and the occurrence of metastasis were microscopically diagnosed. The pretracheal lymph nodes that are detected in front of the central neck are referred to as sub-DLN, and DLN dissections that were performed at our hospital also included dissections of lymph nodes that were located in front of the thyroid cartilage and cricoid cartilage. Surgeries were performed following a typical thyroid dissection method and level VI dissection was performed in all patients, including dissections of pretracheal lymph nodes, paratracheal lymph nodes, and DLN. Based on pathological results, we investigated the relationship between the presence of DLN metastasis and age, tumor size, invasion of the surrounding tissues of the thyroid, lymphovascular invasion, number of central LNMs and the number of lateral neck LNMs. To further evaluate the relationship between DLN metastasis and lateral neck LNM, we also included cases in whom lateral neck lymph node dissection was performed. The central neck lymph node classification was assigned, excluding DLN, to investigate the relationship with further metastasis in the central compartment. The Chi-square test and Fisher’s exact test were used for statistical analysis using SPSS 17.0(SPSS Inc., Chicago, IL, USA) and test results were considered statistically significant when the probability was less than 0.05. The extent of correlation between variables was confirmed using a partial correlation analysis.

## Results

Among 413 patients who underwent thyroid dissection and cervical lymph node dissection due to the presence of PTC, 187 patients (45.3%) were found to have cervical LNM. The DLN was observed in 308 patients (74.6%) and the mean number of DLNs was 2.2. DLN metastasis was observed in 53 patients (17.2%), corresponding to 12.8% of DLN metastases in a total of 413 patients. The mean number of metastasized DLNs was 1.4, as shown in Table 1.

**Table 1:** The rate of detection and metastasis of Delphian lymph nodes (n = 413)

Lymph node metastasis	187 (187/413, 45.3%)
Delphian lymph node detection	308 (308/413, 74.6%)
Delphian lymph node metastasis	53 (53/308, 17.2%)
Mean no. of Delphian lymph nodes	2.23
Mean no. of Delphian lymph node metastases	1.4

There was no statistically significant difference observed between groups where DLN went detected/undetected in the following categories: age at surgery, sex distribution, multifocality, tumor size, thyroiditis, lymphovascular invasion, capsule invasion, central neck LNM, mean number of central lymph nodes removed, lateral neck LNM, mean number of lateral lymph nodes removed and metastasis. A statistically significant difference was observed (2.02 vs. 1.25, *P* <0.001) only when the mean number of central neck lymph nodes in the two groups was compared, as shown in Table 2.

**Table 2:** Demographics of patients with papillary thyroid cancer with and without Delphian lymph nodes

	**Delphian lymph node existence**		***P-value***
	**Present (n =308)**	**Absent (n =105)**	
Age	46.6 ± 10.9 (18 to 75)	50.0 ± 11.8 (26 to 74)	0.346
<45	133 (43.2%)	35 (33.3%)	
≥45	175 (56.8%)	70 (66.7%)	
Sex (F/M)	263/45	90/15	0.926
Multifocality	1.15 (1 to 11)	1.03 (1 to 7)	0.534
Tumor size (mm)	11.37 ± 7.39 (0.5 to 58)	11.43 ± 9.44 (1.5 to 80)	0.864
≤1	161 (52.3%)	60 (58.3%)	
>1	147 (47.7%)	45 (42.9%)	
Thyroditis			0.052
Present	106 (34.4%)	25 (23.8%)	
Absent	202 (65.6%)	80 (76.2%)	
Lymphovascular invasion			0.776
Present	62 (20.1%)	19 (18.1%)	
Absent	246 (79.9%)	86 (81.9%)	
Capsule invasion			0.479
Present	184 (59.7%)	57 (54.3%)	
Absent	124 (40.3%)	48 (45.7%)	
Central metastasis			0.305
Present	140 (45.5%)	42 (40.0%)	
Absent	168 (54.5%)	63 (60.0%)	
Mean no. of central nodes removed	12.08 (0-35)	8.83 (1-51)	0.097
Mean no. of central node metastases	2.02 (0-25)	1.25 (0-8)	0.001
Lateral metastasis			0.098
Present	51 (16.2%)	10 (9.5%)	
Absent	257 (83.8%)	95 (90.5%)	
Mean no. of lateral nodes removed	19.16 (1 to 59)	23.85 (3 to 71)	0.812
Mean no. of lateral node metastases	5.0 (0 to 36)	3.54 (0 to 9)	0.232

Type of surgery in patients with Delphian lymph nodes were Total thyroidectomy with CCND (213/308, 69.1%), Lobectomy with isthmectomy with CCND (44/308, 14.3%), Total thyroidectomy with CCND with MRND (51/308, 16.6%), as shown in Table 3.

**Table 3:** Type of surgery in patients with Delphian lymph nodes who underwent surgery for papillary thyroid cancer

**Type of surgery**	**No. of patients (n = 308)**
Total thyroidectomy c CCND	213 (213/308, 69.1%)
Lobectomy c isthmectomy c CCND	44 (44/308, 14.3%)
Total thyroidectomy c CCND c MRND	51 (51/308, 16.6%)

When cases with DLN metastasis were compared to cases without, univariate analyses showed a statistically significant difference in the following variables: multifocality (1.8 vs. 1.0, *P* <0.001), tumor size larger than 1 cm (66.0% vs. 44.0%, *P* <0.003), lymphovascular invasion (56.6% vs. 12.5%, *P* <0.001), capsule invasion (79.2% vs. 51.3%, *P* <0.001), central neck LNM (88.6% vs. 34.5%, *P* <0.001) and lateral neck LNM (45.3% vs. 10.2%, *P* <0.001), as shown in Table 4.

**Table 4:** Univariate and multivariate analysis of Delphian lymph node metastasis in patients with papillary thyroid cancer

	**Delphian lymph node metastasis**		**Univariate analysis**	**Multivariate analysis**	
	**Present (n = 53)**	**Absent (n = 255)**	***P*-value**	***P*-value**	**CI**
Age	44.7 ± 12.1 (19 to 73)	46.9 ± 10.6 (18 to 75)	0.175		
<45	29 (54.7%)	104 (40.8%)			
≥45	24 (45.3%)	151 (59.2%)			
Multifocality	1.8 (1 to 11]	1.0 (1 to 10]	0.001	0.268	−0.034 to approximately 0.009
Tumor size(mm)	15.7 ± 10.4 (2 to 58)	10.5 ± 6.3 (0.5 to 38)	0.003	0.135	−0.010 to approximately 0.009
≤1	18 (34.0%)	143 (56.0%)			
>1	35 (66.0%)	112 (44.0%)			
Thyroditis			0.169		
Present	14 (26.4%)	91 (35.7%)			
Absent	39 (73.6%)	164 (64.3%)			
Lymphvascular Invasion			0.001	0.005	0.047 to approximately 0.262
Present	30 (56.6%)	32 (12.5%)			
Absent	23 (43.4%)	223 (87.5%)			
Capsule Invasion			0.001	0.849	−0.066 to approximately ~0.080
Present	42 (79.2%)	131 (51.4%)			
Absent	11 (20.8%)	124 (48.6%)			
Central metastasis	53	88	0.001	0.001	0.157 to approximately 0.339
Contralateral central metastasis	28 (52.8%)	36 (14.1%)			
Ipsilateral central metastasis	19 (35.8%)	52 (20.4%)			
Non-metastasis	6 (11.3%)*	167 (65.6%)			
Lateral metastasis			0.005	0.016	0.026 to approximately 0.250
Present	25 (47.2%)	26 (10.2%)			
Absent	28 (52.8%)	229 (89.8%)			

*six patients only Delphian lymph node metastasis.

When multivariate analyses were performed, statistical significant differences were found in lymphovascular invasion (56.6% vs. 12.5%, *P* <0.005), central neck LNM (88.6% vs. 34.5%, *P* <0.001) and lateral neck LNM (47.2% vs. 10.2%, *P* <0.016). However, no statistically significant difference was observed for capsule invasion (79.2% vs. 51.3%, *P* = 0.849), tumor size larger than 1 cm (66.0% vs. 44.0%, *P* = 0.135), multifocality(1.8 vs. 1.0, *P* = 0.268), age (44.7 vs. 46.9, *P* = 0.175) and thyroiditis (26.4% vs. 35.7%, *P* = 0.169), as shown in Table 4.

Central neck LNM was detected in 47 among 53 cases of DLN metastasis (88.6%), while central LNM was observed in 88 of 255 (34.5%) patients without DLN metastasis; a significant difference (*P* <0.001). In other words, the presence of DLN metastasis was associated with an 11.7-fold higher frequency of central neck LNM compared to cases without DLN metastasis. In addition, the number of DLN metastases was correlated with central neck LNM metastasis (correlation coefficient = 0.656, *P* <0.01). Lateral neck LNM was detected in 25 of 53 DLN metastasis cases (47.2%), a proportion that was significantly different compared to cases of lateral neck LNM in which DLN metastasis was not detected (26 of 255 cases, 10.2%; *P* <0.001). This indicates that the presence of DLN metastasis is associated with a 4.4-fold higher frequency of lateral neck LNM compared to when DLN metastasis was absent (Table 5).

**Table 5:** Ability of Delphian lymph node metastasis to predict further central, lateral nodal metastasis

	**Sensitivity**	**Specificity**	**PPV**	**NPV**	**Positive likelihood ratio**	**Negative likelihood ratio**
Central node metastasis	35	97	89	67	11.7	0.69
Lateral node metastasis	48	89	45	90	4.4	0.59

When patients who underwent lateral neck lymph node dissection were excluded, contralateral central neck LNM was detected in 13 of 31 (41.9%) patients with DLN metastasis and in 22 of 226 (9.7%) patients without (Table 6). Considering contralateral central neck LNM depending upon the presence of DLN metastasis, analysis revealed that the probability of not having contralateral central neck LNM in the absence of DLN metastasis absent (negative predictive value (NPV)) was found to be 90.3% (204/226). In particular, when only tumors diameter 1 cm or smaller than 1 cm in size were analyzed, the NPV reached 93.3% (127/136), as shown in Table 7.

**Table 6:** Ipsilateral or contralateral central lymph node metastasis in patients with Delphian lymph node metastasis (excluding those that underwent lateral neck node dissection)

	**Regardless of tumor size Delphian lymph node metastasis**		***P-value***	**Tumor size ≤1 cm Delphian lymph node metastasis**		***P*-value**
	**Present (n = 31)**	**Absent (n = 226)**		**Present (n = 12)**	**Absent (n = 136)**	
Contralateral central LNM	13 (41.9%)	22 (9.7%)	0.001	5 (41.7%)	9 (6.6%)	0.001
Ipsilateral central LNM	12 (38.7%)	40 (17.7%)		2 (16.7%)	20 (14.7%)	
Non-central LNM	6 (19.4%)*	164 (72.6%)		5 (41.7%)*	107 (78.7%)	

*Only Delphian lymph node metastasis.

**Table 7:** Ability of the Delphian lymph node metastasis to predict contralateral central nodal disease

	**Sensitivity**	**Specificity**	**PPV**	**NPV**	**Positive likelihood ratio**	**Negative likelihood ratio**
Contralateral central LNM	37	92	45	90	4.6	0.68
Contralateral central LNM (tumor size ≤1 cm)	35	95	45	93	7.0	0.68

## Discussion

PTC is most frequently observed among thyroid cancers. It generally grows slowly, but requires careful surgery and follow-up observation after the surgery, since many cases show aggressive LNM. Since LNM is frequently observed in PTC, many surgeons perform central neck lymph node dissection on the affected side as a preventive measure [22-24]. In addition, since LNMs were observed in papillary thyroid microcarcinoma (PTMC) 1 cm or smaller than 1 cm in size, many studies have stressed the importance of monitoring for lymph node recurrence [7,13,22,25-27]. Previously, the effect of cervical LNM on survival rate for PTC was reported to be low [5], but recent studies have reported significant associations between LNM and loco-regional recurrence and survival rate [1,3,6-11].

Under such circumstances, attention has been directed toward the clinical role of DLN in thyroid cancers, and Isaacs *et al.* reported the probabilities of central neck LNM and lateral neck LNM in the presence of DLN metastasis in level VI lymph nodes to reach 85% and 83%, respectively. The authors of that study also proposed that the presence of central neck LNM is predictive of thyroid cancer progression, and claimed that the presence of DLN metastasis is the most useful predictive factor [16]. In another study, Isaacs *et al.* also reported the detection rate of DLN in PTC patients to be 48.3% (87/180), lower than the 74.5% (308/413) discovered in the present study, but they reported a DLN metastasis rate of 20.7% (18/87), closer to the 17.4% reported here [15].

Iyer *et al.* reported that DLN metastasis in thyroid cancers is associated with extrathyroidal extension (ETE) and the necessity of careful lymph node dissection and frozen section biopsy during surgery because its presence is correlated with central neck LNM and lateral neck LNM [20]. In the present study, the DLN metastasis group had a significantly higher proportion of patients with lymphovascular invasion (56.6% vs. 12.5%, *P* <0.005), further central neck LNM (88.6% vs. 34.5%, *P* <0.001), and lateral neck LNM (47.2% vs. 10.2%, *P* <0.016) than the group in which DLN metastasis was absent. The frequency of central neck LNM was 88.6% (47/53) in the group with DLN metastasis, and DLN metastasis was also significantly correlated with central neck LNM (correlation coefficient = 0.656, *P* <0.01). These findings indicate that total thyroidectomy and central neck lymph node dissection is the most appropriate treatment choice in patients with DLN metastasis.

Ito *et al.* reported that central neck LNM is not associated with disease-free survival, and PTMC limited to one side of the lobe may not perform central neck lymph node dissection in the contralateral lobe in select cases [5,8,13,24,27]. Roh *et al.*[(2008)] investigated the metastasis pattern of central neck lymph nodes in PTMC and reported that ipsilateral paratracheal LNM was observed in 27 of 72 cases (37.5%) and contralateral paratracheal LNM was observed in 1 of 57 cases (1.7%) [27]. In the present study, if DLN metastasis was absent, the NPV for contralateral central neck LNM was found to be 90.3% (204/226). The NPV for LNMs tumor sized 1 cm or smaller than 1 cm was 93.3% (127/136), as shown in Table 7. These results suggest that performing thyroid lobectomy and ipsilateral central neck lymph node dissection as a consequence of frozen section biopsy results during surgery, in addition to limiting contralateral central neck lymph node dissection to select cases, could be sufficient for treating PTC patients.

Wada *et al.* (2003) reported a high recurrence rate of lateral neck lymph node metastasis if lymph nodes were visible with the naked eye during surgery (16.7% vs. 0.43%)^1^. In laryngeal cancer, the presence of DLN metastasis increased lateral neck LNM, resulting in a high recurrence rate and low survival rate. The presence of DLN metastasis in laryngeal cancer is known to be a significant prognostic factor [18,19,21]. Delbridge *et al.* reported that the probability of having lateral neck LNM when DLN metastasis was present in thyroid cancer patients reached 74%, and proposed that DLN metastasis should be used as a predictor of N1b stage [15,16]. In the present study, the proportion of patients who experienced lateral neck lymph node metastasis was significantly higher in the DLN metastasis group (25 of 53 cases (47.2%)) than in the group in which DLN metastasis was not detected (26 of 255 (10.2%); *P* <0.001). The presence of DLN metastasis was associated with a 4.4-fold higher frequency of lateral neck LNM. Therefore, if DLN metastasis is detected after surgery (even if lateral neck LNM was not detected on preoperative imaging), we recommend vigilance for the recurrence of lateral neck LNM during follow-up.

## Conclusion

Since the presence of central neck LNM seems to be associated with DLN metastasis, total thyroidectomy and central neck lymph node dissection should be the first-line treatment in patients with DLN metastasis. In contrast, when DLN metastasis is not observed in PTMC, thyroid lobectomy on the affected side and ipsilateral central neck lymph node dissection should be sufficient. A retrospective investigation of the presence of central neck LNM and metastasis pattern with respect to the presence of DLN metastasis in patients who underwent total thyroidectomy may yield more useful results for planning the extent of surgery. In addition, if DLN metastasis is detected after surgery (even if lateral neck LNM was not detected on preoperative imaging), the possibility of lateral neck LNM recurrence during follow-up should be considered.

## Consent

Informed consent was omitted because of the retrospective character of the study and approval was given by the Kosin University Gospel Hospital institutional review board.

## Abbreviations

CCND: Central compartment neck dissection; DLN: Delphian lymph node; ETE: Extra-thyroidal extension; LNM: Lymph node metastasis; MRND: Modified radical neck dissection; NPV: Negative predictive value; PPV: Positive predictive value; PTC: Papillary thyroid cancer; PTMC: Papillary thyroid microcarcinoma.

## Competing interests

The authors declare that they have no competing interests.

## Authors’ contributions

JHK, the corresponding author of this study, provided the major idea, planed and approved the written work, performed the operations. WWK contributed literature review and writing the manuscript and analyzing clinicopathologic data. SIY contributed literature review and drafted the manuscript. YSC, YHP and SKK gave advices about clinical variables to analyze and edited the discussion. All authors read and approved the manuscript.
